# Single-walled carbon nanotubes modulate pulmonary immune responses and increase pandemic influenza a virus titers in mice

**DOI:** 10.1186/s12985-017-0909-z

**Published:** 2017-12-22

**Authors:** Hao Chen, Xiao Zheng, Justine Nicholas, Sara T. Humes, Julia C. Loeb, Sarah E. Robinson, Joseph H. Bisesi, Dipesh Das, Navid B. Saleh, William L. Castleman, John A. Lednicky, Tara Sabo-Attwood

**Affiliations:** 1 Department of Environmental and Global Health, Center for Environmental and Human Toxicology and Emerging Pathogens Institute, University of Florida, 2187 Mowry Road, Box 110885, Gainesville, FL 32611 USA; 2 Department of Physiological Sciences, 1333 Center Drive, Box 100144, Gainesville, FL 32610 USA; 3 Department of Department of Civil, Architectural, and Environmental Engineering, University of Texas Austin, Austin, TX 78712 USA; 4 Department of Infectious Diseases and Pathology, PO Box 110880, Gainesville, FL 32611 USA

**Keywords:** Single-walled carbon nanotubes, Influenza A virus, H1N1, C57BL/6 mice, Infectivity, Cytokines, Interferon-induced protein with tetratricopeptide repeats, Pathogen

## Abstract

**Background:**

Numerous toxicological studies have focused on injury caused by exposure to single types of nanoparticles, but few have investigated how such exposures impact a host’s immune response to pathogen challenge. Few studies have shown that nanoparticles can alter a host’s response to pathogens (chiefly bacteria) but there is even less knowledge of the impact of such particles on viral infections. In this study, we performed experiments to investigate if exposure of mice to single-walled carbon nanotubes (SWCNT) alters immune mechanisms and viral titers following subsequent influenza A virus (IAV) infection.

**Methods:**

Male C57BL/6 mice were exposed to 20 μg of SWCNT or control vehicle by intratracheal instillation followed by intranasal exposure to 3.2 × 10^4^ TCID_50_ IAV or PBS after 3 days. On day 7 mice were euthanized and near-infrared fluorescence (NIRF) imaging was used to track SWCNT in lung tissues. Viral titers, histopathology, and mRNA expression of antiviral and inflammatory genes were measured in lung tissue. Differential cell counts and cytokine levels were quantified in bronchoalveolar lavage fluid (BALF).

**Results:**

Viral titers showed a 63-fold increase in IAV in SWCNT + IAV exposed lungs compared to the IAV only exposure. Quantitation of immune cells in BALF indicated an increase of neutrophils in the IAV group and a mixed profile of lymphocytes and neutrophils in SWCNT + IAV treated mice. NIRF indicated SWCNT remained in the lung throughout the experiment and localized in the junctions of terminal bronchioles, alveolar ducts, and surrounding alveoli. The dual exposure exacerbated pulmonary inflammation and tissue lesions compared to SWCNT or IAV single exposures. IAV exposure increased several cytokine and chemokine levels in BALF, but greater levels of IL-4, IL-12 (P70), IP-10, MIP-1, MIP-1α, MIP-1β, and RANTES were evident in the SWCNT + IAV group. The expression of *tlr3, ifnβ1, rantes, ifit2, ifit3, and il8* was induced by IAV alone but several anti-viral targets showed a repressed trend (*ifits*) with pre-exposure to SWCNT.

**Conclusions:**

These findings reveal a pronounced effect of SWCNT on IAV infection in vivo as evidenced by exacerbated lung injury, increased viral titers and several cytokines/chemokines levels, and reduction of anti-viral gene expression. These results imply that SWCNT can increase susceptibility to respiratory viral infections as a novel mechanism of toxicity.

**Electronic supplementary material:**

The online version of this article (10.1186/s12985-017-0909-z) contains supplementary material, which is available to authorized users.

## Background

Rapid advancement in nano-scale material synthesis has led to the development of numerous and diverse types of nanomaterials (NM) that are being widely utilized in the industrial and consumer product sectors [1]. One type of NM, carbon nanotubes (CNT), have strong carbon-carbon (C-C) sigma bonds and sp2 hybridization that form nanoparticles with high tensile strength and superior thermal and electrical properties [2]. Such properties have led to the use of CNT in electrodes, supercapacitors, catalysis, filters and membranes, sensors, and are being explored for biomedical applications [3–5]. The implementation of CNT-related products, research publications, and patents continues to increase and De Volder and colleagues report the worldwide production capacity of CNT has increased more than 10 fold since 2006 [3].

Potential environmental exposures to CNT is likely to arise through release during various stages of product lifecycles that include manufacturing, product use, end-of-life and waste leaching through ingestion, inhalation, and direct dermal contact [6]. Due to their ultra-small size and based on our knowledge of particulate deposition in the respiratory tract, CNT are accessible to deeper parts of the airway, supporting inhalation as a critical route of exposure [7, 8]. Detectable levels of CNT have been recently measured in air and surfaces in industrial settings where these materials are being utilized in manufacturing products and adverse biological effects have been found among these workers [9–11]. Moreover, several in vivo studies report that different types of CNT can induce varied adverse pulmonary effects including sub-chronic tissue damage, fibrogenesis, granulomatous changes, impaired clearance, robust inflammation, airway hyper-reactivity, and cardiopulmonary consequences, among others [12–14]. Few studies, however, have investigated whether pulmonary exposure to CNT alters the hosts’ susceptibility to subsequent pathogen infections, particularly respiratory viruses. In fact, our group is the first to demonstrate that CNT modulate typical cellular responses to influenza virus resulting in enhanced infectivity [15]. Specifically, we reported that pre-exposure of human small airway epithelial cells to single-walled carbon nanotubes (SWCNT) results in significant increases in pandemic influenza A virus H1N1 (IAV) titers [15]. Noteworthy, these particular lung cells are found in the distal portion of the human respiratory tract and IAV infections of the lower respiratory tract are associated with increased disease severity [16], a scenario that is likely true for other respiratory pathogens.

Several lines of epidemiological evidence support that inhalation of particulates generated in the workplace or ambient environment result in increased incidence and severity of respiratory infections that carry significant health and economic burden [17]. In the U.S. alone, upper respiratory infections caused by non-influenza viruses are responsible for 500 million infections annually, a loss of 40 billion dollars and 40–100 million school and work days [18]. Populations exposed to particulates, such as those present in ambient air pollution (PM_2.5_, diesel exhaust particulates, secondary organic aerosols), and the workplace (silica, particulates in welding fumes) are reported to have higher infection rates and increased emergency room visits [19–21]. Similar in size and structure to some of these particulates, CNT are suspected to possess the same ability to exacerbate pathogenic infections. A few studies have reported that inhalation of CNT can inhibit bacteria phagocytosis and clearance with enhanced pulmonary inflammation and fibrosis [2, 22, 23]; sequential exposure to SWCNT and *Listeria monocytogenes* enhanced pulmonary inflammation and infectivity in mice [22]; and helical CNT enhanced the immune response and inhibited macrophage-mediated phagocytosis of *Pseudomonas aeruginosa* [2]. In a more recent study, NM were shown to reactivate latent herpesvirus in mouse and human cells [24].

While these studies provide a rationale for investigating the impact of CNT on pathogenic infections, we are the only group to date that has investigated how SWCNT increase IAV infections specifically. Our previous work provided evidence that SWCNT inhibited IAV-induced expression of several antiviral and inflammatory markers (CCL5, IFIT2, IFIT3) [15] but these observations were limited to in vitro studies in lung epithelial cells. Here we investigated the effect of SWCNT exposure on subsequent pulmonary infection with IAV, assessing viral titers, antiviral, and immune responses in an in vivo mouse model. Overall, we report that pre-exposure to SWCNT results in significant enhancement of IAV titers while exacerbating lung tissue inflammation and enhanced expression of several relevant cytokines and chemokines.

## Methods

### SWCNT suspension preparation

Pristine SG65i SWCNT were provided by SouthWest Nanotechnologies Inc. (SWeNT). The SG65i are a mixture of primarily semi-conductive and chirality-enriched non-functionalized CNTs characterized by our group, measuring 0.78 nm in diameter and <4% cobalt and molybdenum [15]. All SWCNT suspensions were prepared in 2% sodium deoxycholate (SDC) and dialyzed in 1% pluronic F68 (*v*/v in deionized water, Sigma) using protocols similar to methods previously described [25] to maintain innate fluorescent properties. Briefly, dry SG65i were suspended with 2% SDC to 1 mg/mL and sonicated (Sonifier™ S-450 Digital Ultrasonic Cell Disruptor/Homogenizer, Branson Ultrasonics, Danbury, CT) at 30–50 watt power for 20 min in an ice bath. SWCNT were then diluted in 2% sodium cholate, and their absorbance read at 775 nm (SOFTmax Pro4.0, Synergy H1, Biotek) [15]. The remaining SWCNT stock was centrifuged at 14,100 rpm for 20 min and then supernatant was collected and sonicated at 30 watts for 10 min. The suspension was transferred into 3 mL dialysis catridges (3500 MWCO, Thermo-Scientific) which were soaked in 1% pluronic for surfactant exchange. The dialysis process continued for 3 consecutive days and the absorbance measured at the end of dialysis. Final concentration of SWCNT was calculated as follows: starting concentration × (after dialysis absorbance/pre-dialysis absorbance). The SWCNT suspension was re-sonicated at 30 watts for 1 min before use.

### SWCNT suspension characterization

Time dependent hydrodynamic radii (HDR) of SDC and pluronic modified SWCNT in water were monitored with time resolved dynamic light scattering (TRDLS). The SWCNT concentration for the DLS measurements was 10 mg/L. A 22 mW 632 nm HeNe laser incorporated ALV/CGS-3 compact goniometer system (ALV-Laser GmbH, Langen/Hessen, Germany) with QE APD detector (photomultipliers of 1:25 sensitivity) was employed to monitor size evolution every 15 s for 48 h. The TRDLS experiments were performed at 37 °C with 2 mL sample in a borosilicate glass vial, which was cleaned thoroughly with 2% extran solution. The vial was vortexed for 10s before inserting in the toluene-filled sample vet of the goniometer system. The scattered light was collected at 90° and analyzed using an auto cross-correlator to calculate average HDR. The electrophoretic mobility (EPM) of SWCNT suspensions was measured using a Möbiuζ (Wyatt Technology, Santa Barbara, CA) at 25 °C and 30 psi. For each measurement, 1 mL of the suspension was introduced into a flow through cell using a 1 mL syringe. Five EPM values were collected for each suspension.

### Influenza virus preparation and titration

Influenza virus H1N1 strain A/Mexico/4108/2009 was kindly provided by Drs. Gary Heil and Gregory Gray and was propagated in MDCK cells in serum-free medium with 1 or 0.1 μg/ml L-1-tosylamide-2-phenylethyl chloromethyl ketone (TPCK)-treated trypsin as previously described [15]. To quantify the number of virus particles we calculated TCID_50_ values using a standard assay [15]. Briefly, part of the right lung tissue of each mouse was weighed and homogenized using disposable tissue grinders (VWR, Radnor, PA) in advanced DMEM media (Invitrogen, Carlsbad, CA) containing 1% penicillin-streptomycin-neomycin antibiotic mixture (Fisher, Waltham, MA) and 1% L-Glutamax (Fisher, Waltham, MA). To perform the titer assays, MDCK cells were seeded in 96 well plates and serial dilutions (10^−1^, 10^−2^, 10^−3^, 10^−4^, 10^−5^, 10^−6^) of virus stock and lung tissue homogenates were added to cell monolayers. Cells were incubated at 33 °C and monitored for cytopathic effects (CPE) by light microscopy 5 days post infection. Each well that displayed CPE was scored as positive. The TCID_50_ was calculated as the dilution of virus at which 50% of the cell cultures were infected.

### Animals

Male C57BL/6 mice were obtained from the Jackson Laboratory (Bar Harbor, ME) and housed in plastic cages and provided with water and feed ad libitum at 25 °C, 50% humidity and 12/12 h light/dark cycle at the Animal Care Service Facility. All animals were acclimated in ambient conditions for a week before any treatment were given. The mice were treated humanely according to the NIH *Guide for the Care and Use of Laboratory Animals* and all protocols were approved by the Institutional Animal Care and Use Committee (IACUC) at the University of Florida.

### Preliminary IAV dose finding study

Twenty mice at the age of 12 weeks were randomly assigned into four groups (*N* = 5). On day 0, animals were anesthetized with a cocktail of ketamine (100 mg/kg x bw) and xylazine (5 mg/kg x bw) (Patterson Veterinaries Inc., Charlotte, NC) through intraperitoneal injection. After anesthesia, the mice were given 50 μL of different doses of IAV (10^0^, 10^2^, and 10^4^ TCID_50_ per animal) or phosphate-buffered saline (PBS) through intranasal aspiration. Daily body weight and clinical signs of infection were monitored daily. Mice were euthanized on days 3 and 7 with intraperitoneal injection of 0.2 mL fatal plus solution (VorTech Pharmaceuticals). Mouse lungs were collected and frozen in liquid nitrogen for virus titration.

### Sequential exposures of SWCNT and virus to mice

Forty mice at the age of 12 weeks were randomly assigned into four groups (*N* = 10) control, SWCNT only, IAV only, and SWCNT + IAV. Mice were anesthetized as described above prior to positioning each animal on a rodent-tilting work station and inoculated with 40 μL of either SWCNT (20 μg per animal) or control vehicle at the entrance of the trachea on day 0. On day 3, animals received 50 μL of either IAV (3.2 × 10^4^ TCID_50_ per animal) or PBS through intranasal aspiration. Animals were weighed and monitored for clinical signs daily. On day 7, all animals were euthanized with 0.2 mL fatal plus solution.

### Immune cell identification in bronchoalveolar lavage fluid (BALF)

Following euthanasia, mouse lungs were lavaged with 1 mL cold PBS and the recovered fluid was collected and immediately placed on ice. BALF volumes were recorded and the total number of cells quantified by counting on a haemocytometor. The volume of BALF required for 25,000 cells total per sample was spun onto glass slides using a cytospin (ThermoFisher). All slides were stained using a HEMA III kit (ThermoFisher) and 200 cells per slide were counted and identified as monocytes, lymphocytes, neutrophils, eosinophils, or basophils. All samples were counted duplicate and the average values were calculated.

### Histopathology and IAV immunohistochemistry

The left lung of each animal was perfused with freshly prepared 4% paraformaldehyde (PFA) in PBS at pH 7.4, then excised and stored in the same PFA solution. Fixed lung tissues were embedded in paraffin wax and sectioned at approximately 5 μm. Sections were then deparaffinized and stained with hematoxylin and eosin (H&E) for histopathologic and morphometric analysis by a certified pathologist (W.L.C.). Each of 2 lung sections from the left lung of each mouse will be assessed for bronchitis, bronchiolitis, and interstitial pneumonia. In addition to qualitative histopathology assessment, volume density of pneumonia will be calculated by digitizing morphometry as described [26]. Density of H1N1 viral antigen-positive cells in area of pneumonia and percentage of viral antigen-positive bronchioles will be calculated [27]. Additional paraffin sections for H1N1 antigen immunohistochemistry will be mounted on charged slides, de-paraffinized and rehydrated prior heat-induced epitope retrieval in Tris-EDTA, pH 9 at 95 °C for 20 min. Endogenous peroxidase activity will be quenched by treatment with 0.3% H_2_O_2_ in methanol for 30 min. Sections will be treated with horse blocking serum prior to incubation with goat anti-H1N1 (AB1074, Millipore, Billerica, MA) at 1:500. Bound antibody will be detected with an ImmPRESS anti-goat IgG-polymer detection kit (MP-7405, Vector Laboratories, Burlingame, CA) prior to peroxidase activity detection with Metal Enhanced DAB (3,3′-diaminobenzidine) Substrate Working Solution (Thermo Scientific, Rockford, IL) as described in the kit. Slides will be lightly stained with Harris hematoxylin.

### Near infrared fluorescence (NIRF) imaging of SWCNT in lung tissue

H&E stained mouse lung sections were imaged using a custom NIRF system and the fluorescent signals were captured and processed by WinView software (Princeton Instruments, Trenton, NJ). All pictures presented were background subtracted and standardized (intensity range) as previously described [25]. Final NIRF images were merged over corresponding brightfield pictures in Photoshop (Adobe).

### Quantitation of cytokines and chemokines in BALF

Expression levels of 25 cytokines and chemokines (G-CSF, GM-CSF, IFNγ, IL-1α, IL-1β, IL-2, IL-4, IL-5, IL-6, IL-7, IL-9, IL-10, IL-12 (p40), IL-12 (p70), IL-13, IL-15, IL-17, IP-10, KC, MCP-1, MIP-1α, MIP-1β, MIP-2, RANTES, and TNFα) were quantified in BALF using the MILLIPLEX mouse cytokine / chemokine magnetic bead panel (Millipore) according to the manufacturer’s protocol. The samples were acquired on Luminex 200 system (Luminex) and the amount (ng/mL) was calculated using xPONENT software (Luminex).

### Expression levels of immune- and anti-viral related genes in lung tissue

Changes in mRNA expression in SWCNT- and IAV-exposed mouse lungs was performed by real-time qRT-PCR as previously described [28]. Briefly, frozen lung tissues (from the lavaged right lung lobe) were homogenized with RNA Stat-60 (Tel-Test) following manufacturer’s instructions. RNA pellets were reconstituted in RNA secure (Ambion) and incubated for 10 min at 60 °C to inactivate RNases. The resultant cDNA was amplified using validated primers (Additional file 1: Table S2) and probes specific to each gene target; *ccl5, il8, ifit2. ifit3, ifnβ1, tlr3* (Applied Biosystems). Expression of GAPDH was employed for a standard housekeeping gene and all data is presented as normalized fold change in expression compared to controls using the ∆∆Ct method.

### Statistical analysis

One-way ANOVA was used to compare the average mean of parameters described above unless indicated specifically among different treatment groups. Pair-wise comparisons were conducted by using Tukey’s test when equal variances were confirmed and Dunnett’s test for unequal variances. T-test was used to compare the difference of viral titer between IAV only group and SWCNT + IAV groups. Data were tested for normal distribution for one-way ANOVA and t-test. Data with non-normal distribution would be transformed to achieve normal distribution. Due to unequal sample size and non-normal distribution of data, immune cell counts and gene expression data were compared by using Kruskal-Wallis rank-sum test and pair-wise comparison were conducted by using Dunn’s multiple comparison test. Statistical differences were identified at *P* < 0.05.

## Results

### Influenza A virus dose finding study in mice

**Fig. 1:**
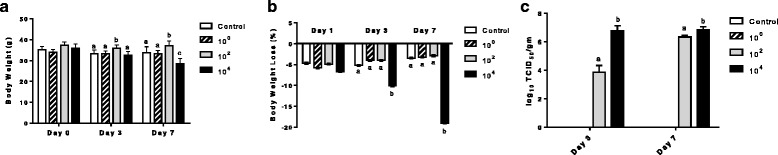
IAV dose finding in mice. C57BL/6 mice were exposed to different doses of IAV (0, 10^0^, 10^2^, 10^4^ TCID_50_ per mouse) on day 0 and mice were euthanized on day 3 or 7, respectively. **a** Body weight was recorded on days 0, 3, and 7 as well as **b** body weight loss on days 1, 3, and 7. Data are presented as mean±SEM. **c** Virus titers were quantified in lung homogenates by TCID_50_ assay. Data are presented as mean±SEM of Log_10_ transformed TCID_50_ / mL. Letters indicate significant differences between groups (*P* < 0.05)

To determine a dose and exposure duration of IAV that caused moderate illness in C57BL/6 mice, each animal was exposed to vehicle control (PBS), 10^0^, 10^2^, or 10^4^ TCID_50_ IAV particles of pandemic influenza A/Mexico/4108/2009 (H1N1) through intranasal administration. Body weight (g) was recorded daily and viral titers (TCID_50_) were quantified from lung tissues collected on day 7 post infection. Results in Fig. 1 show that a significant reduction in body weight occurred only for the highest dose of IAV (10^4^ TCID_50_/mouse) by 3 days (10% loss) and reached 19% by 7 days (Fig. 1B). Virus titers plateaued at 10^7^ TCID_50_/g on day 3 for the highest dose of IAV and remained at that level on day 7. Our findings are consistent with other studies that have observed similar loss in body weight for this particular strain of IAV [29–32]. Based on these initial results, we chose to use a dose of 10^4^ TCID_50_/mouse with an exposure time of 4 days in subsequent studies which would allow us to investigate biological responses with a moderate level of illness.

### SWCNT pre-exposure increased IAV titers in mice

**Fig. 2:**
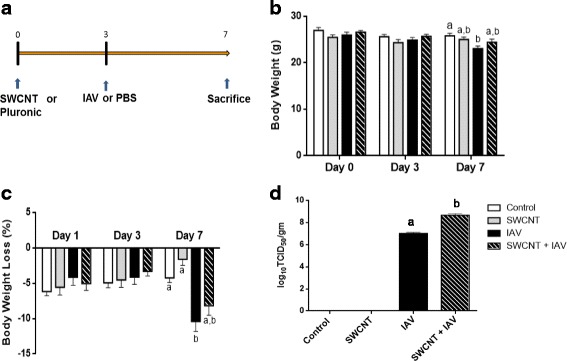
Exposure design and changes in body weight and virus titer in SWCNT and IAV exposed mice. **a** Schematic of exposure design. C57BL/6 mice were exposed to SWCNT (20 μg per mouse) on day 0 and then to IAV (3.2×10^4^ TCID_50_ per mouse) on day 3. On day 7 mice were euthanized and samples were collected for different assays. **b** Total body weight and **c** weight loss were recorded on days 0, 3, and 7. **d** Virus titers were quantified in lung homogenates by TCID_50_ assay. Data are presented as mean±SEM of Log_10_ transformed TCID_50_ / gm. Letters indicate significant differences between groups (*P* < 0.05)

To test whether pre-exposure to SWCNT would alter IAV titers in vivo, mice were first administered SWCNT (20 μg/mouse) by intratracheal instillation followed by exposure to IAV (3.2 × 10^4^ TCID_50_/mouse) (Fig. 2A). The dose of SWCNT was chosen as it falls below those reported in actual workplace conditions and is lower than the current proposed life-time work exposures values [33]. SWCNT suspensions contained agglomerates that primarily ranged from 100 nm – 200 nm with a mean of 151±16.4 nm over a 48-h period at 37 °C. The surface charge was calculated to be slightly negative with an EPM value of −1.43 ± 0.04 × 10^−8^ m^2^ V^−1^S^−1^ (Additional file 1: Figure S1). At the end of the entire exposure period (7 days), mice showed typical signs of IAV infection that included minor labored breathing, reduced activity, and decreased body weight (Fig. 2B) [34]. Body weight was also monitored daily (Additional file 1: Table S1). Across the 7-day period, no significant change was observed in body weight for mice in Control and SWCNT only groups; however, mice in both IAV only and SWCNT + IAV exposed groups showed significantly decreased body weight by day 7 resulting in 10.4% and 8.2% loss, respectively (Fig. 2C).

Viral titers were quantified in mouse lung tissues revealing (Fig. 2D) 10^7^ TCID_50_/g for the IAV only group which was consistent with our dose-finding study (Fig. 1) and previous reports which range from 10^5^ to 10^7^ TCID_50_/g post infection 3–4 days [30–32]. Strikingly, the titer in the co-exposed group of SWCNT + IAV reached 10^9^ TCID_50_/g which was significantly elevated by 63 fold compared to the IAV only group.

### SWCNT and IAV sequential exposure induced a mixed profile of immune cells in bronchoalveolar lavage fluid

**Fig. 3:**
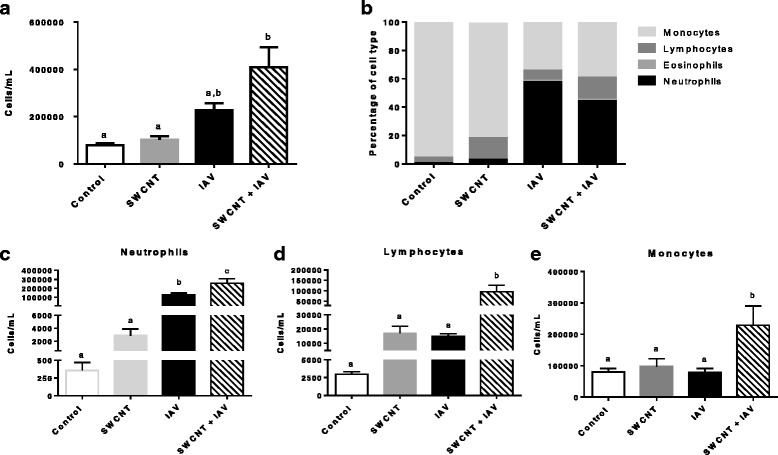
Changes in immune cell profiles in BALF from mice exposed to SWCNT and IAV. C57BL/6 mice were exposed to SWCNT (20 μg per mouse) on day 0 and then to IAV (3.2×10^4^ TCID_50_ per mouse) on day 3. On day 7 mouse lungs were lavaged with PBS and immune cells were counted; **a** total immune cell counts (number / mL), **b** percentage of immune cell types, and numbers of **c** neutrophils **d** lymphocytes and **e** monocytes (number / mL). Results are presented as mean±SEM of 4–6 mice per group. Letters indicate significant differences between groups (*P* < 0.05)

Total cell numbers and differential cell counts in BALF from mice in all treatment groups were quantified. The number of recovered cells showed an increasing trend in IAV only treated mice that was significantly increased in the combined SWCNT + IAV group (Fig. 3A). BALF from Control mice (Fig. 3B) showed a dominating proportion of monocytes whereas IAV exposure markedly shifted the cells to a neutrophil abundant profile. These results are expected and consistent with IAV infections in mice [35, 36]. Surprisingly, BALF from mice exposed only to SWCNT did not show significantly enhanced cell numbers over Control animals although quantitation of specific cell types showed an increased trend in the number of neutrophils (Fig. 3C) and in particular, lymphocytes (Fig. 3D). Interestingly, SWCNT + IAV presented with a mixed immune cell profile that included a significant increase in the number of neutrophils (Fig. 3C), lymphocytes (Fig. 3D) and monocytes (Fig. 3E). Overall, the combined treatment caused the most significant increase in total cell numbers and shifted the population to a ‘mixed’ phenotype reminiscent of the SWCNT and IAV only groups.

### SWCNT and IAV exposure increased pulmonary inflammation and lung injury

**Fig. 4:**
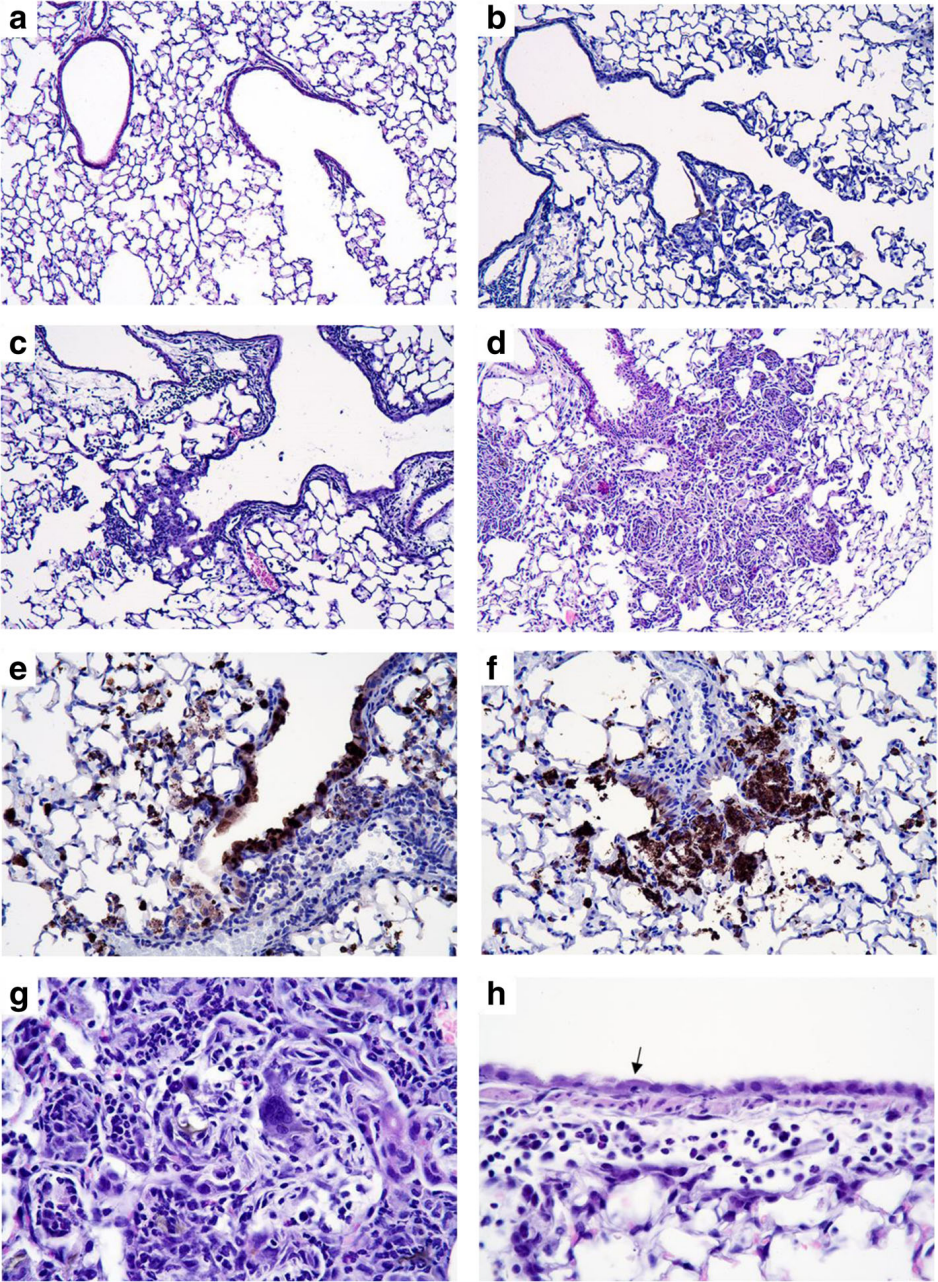
Histopathological changes in mouse lung tissues after exposure to SWCNT and IAV. C57BL/6 mice were exposed to SWCNT (20 μg per mouse) on day 0 and then to IAV (3.2×10^4^ TCID_50_ per mouse) on day 3. On day 7 mouse lungs were excised and paraffin sections were prepared. H&E stained lung sections were analyzed for pulmonary injury under brightfield microscopy (150X) for **a** Control, **b** SWCNT only, **c** IAV only, and **d** SWCNT + IAV groups. Select sections were stained with IAV antigen by immunohistochemistry (300X) for **e** IAV only exposure and **f** SWCNT + IAV. An example of **g** interstitial pneumonia and **h** cuboidal bronchiolar epithelium after SWCNT + IAV exposure are shown (610X)

Lung tissue was examined for histopathological changes and detection of IAV by immunohistochemistry. Exposure to SWCNT alone induced localized lesions of macrophage-dominated pneumonia (Fig. 4B) while IAV alone induced bronchiolitis and patchy interstitial pneumonia in proximal acinar areas (Fig. 4C) compared to Controls (Fig. 4A). These observations are in line with reports showing that SWCNT induced pneumonitis with accumulation of pulmonary neutrophils and macrophages at peribronchiolar regions and alveoli in conjunction with fibrotic and granulomatous lesions 7 days post exposure [22, 37, 38]. Studies also indicated that IAV only exposure was consistently characterized by severe interstitial pneumonia accompanied by mass infiltration of lymphocytes and neutrophils, bronchial lesions, and alveolar damage with edema [34, 39].

**Fig. 5:**
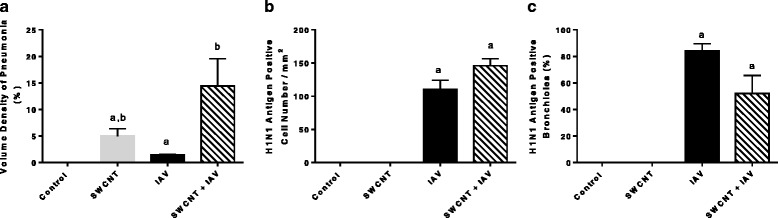
Semi-Quantitation of histopathological changes. C57BL/6 mice were exposed to SWCNT (20 μg per mouse) on day 0 and then to IAV (3.2×10^4^ TCID_50_ per mouse) on day 3. On day 7 lung sections were scored for **a** volume density pneumonia, **b** H1N1 antigen positive cell density in pneumonic area, and **c** H1N1 antigen positive bronchioles. Results are presented as mean±SEM of 4–6 mice per group. Letters indicate significant differences between groups (*P* < 0.05)

Intriguingly, there was overlap in the nature of the lesions induced by the combined injury. More specifically, combined exposure to SWCNT + IAV, caused more severe interstitial pneumonia (Fig. 4D,G) with notable larger areas in proximal acinar areas than in either of the single injuries, causing a higher volume density of pneumonia that was measured by digitizing morphometry (Fig. 5A). However, there were also significant differences in the magnitude of the lesions. In proximal acinar areas with combined injury, in addition to the larger volume of inflammation, there was higher density of inflammatory cells that included multinucleated giant cell macrophages and neutrophils. There were also higher density aggregates of fibroblasts in alveolar walls and in alveoli filled with exudate, and IAV antigen deposits were increased in density in these areas (Fig. 4E,F). IAV antigen was localized in bronchiolar epithelial cells and in type II alveolar epithelial cells and macrophages in areas of pneumonia (Fig. 5B); however, the increased density was not associated with a clearly demonstrable increase in the density of nucleated cells with diffusely antigen positive cytoplasm. The increased density appeared to be accounted for in part by antigen-positive cytoplasmic debris and phagocytosed particulate antigen debris in macrophage cytoplasm. The percentage of bronchioles with epithelium positive for IAV antigen appeared to be lower (*P* = 0.07) than in combined injury mice compared to those only inoculated with IAV (Fig. 5C). This appeared to be associated with more extensive necrosis of bronchiolar epithelium in the combined injury group. The bronchiolar epithelium appears low cuboidal instead of columnar indicating response to injury, and necrotic bronchiolar cells with pyknotic nuclei are noted (Fig. 4H).

### Localization of SWCNT in mouse lung tissue using near infrared fluorescence (NIRF)

**Fig. 6:**
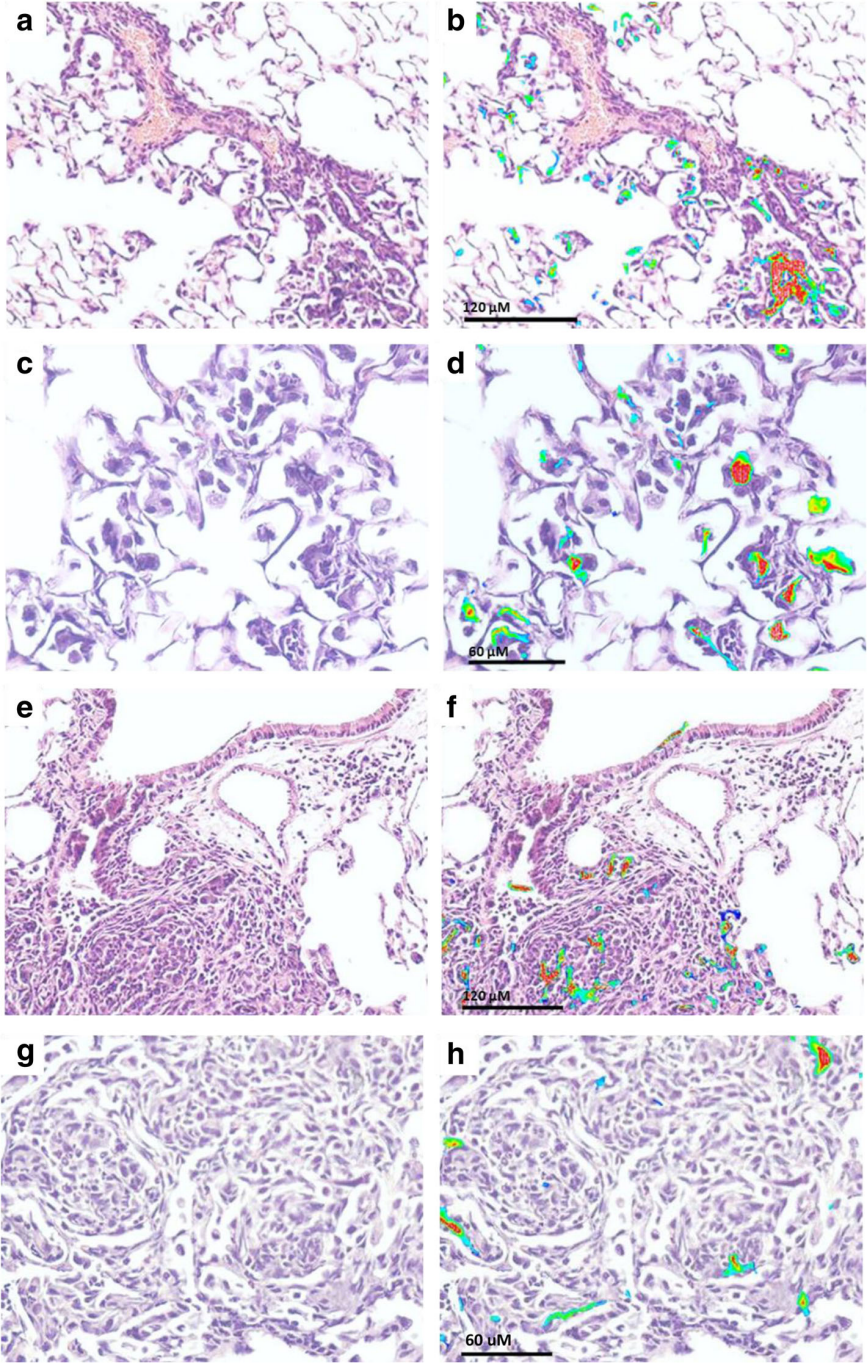
Near infrared fluorescence (NIRF) imaging for tracking SWCNT in mice. C57BL/6 mice were exposed to SWCNT (20 μg per mouse) on day 0 and then to IAV (3.2×10^4^ TCID_50_ per mouse) on day 3. On day 7 mouse lungs were stored in PFA and paraffin sections were prepared. H&E stained sections were probed with NIRF imaging and images aligned. **a**, **c** H&E SWCNT only images; **b**, **d** overlaid H&E and NIRF SWCNT only images; **e**, **g** H&E SWCNT + IAV images; **f**, **h** overlaid H&E and NIRF images for SWCNT + IAV

Due to their innate ability to fluoresce in the near infrared range, the particular SWCNT utilized in the current study can be detected and tracked in tissues using a NIRF method previously optimized by our group [25, 40–42]. NIRF signals on H&E stained mouse lung sections were imaged with a custom laser-based system at 200X and 400X magnification. Lung tissue sections in Control and IAV exposed mice showed no fluorescent background signal as expected (Additional file 1: Figure S2). However, analysis of lung sections from both SWCNT- and SWCNT + IAV-exposed animals produced fluorescent signals that varied in intensity and location (Fig. 6). While fluorescence was matched to obvious “black aggregates” that could be seen in the airways or tissues under brightfield microscopy, there were also prominent signals in areas with no visible presence of SWCNT (i.e. black aggregates). This highlights the utility and strength of using NIRF to localize SWCNT in vivo directly on H&E stained tissue sections that can be analyzed in tandem for pathological injury. Additional strengths of this method are the lack of background fluorescence, sensitivity and false positives that may arise from staining artifacts (such as with TEM).

In general, the NIRF signals were heterogeneously dispersed where nanotubes were visualized in dense areas of immune cell infiltration and pneumonia but also in less dense areas that corresponded to what appears to be individual or clusters of immune and epithelial cells. Interestingly, the dense areas of pneumonia induced by the SWCNT + IAV exposure contained some fluorescence signal but overall the SWCNT did not seem concentrated in these areas where the majority of IAV was detected. It is also worth noting that after 7 days, the SWCNT were still very apparent in the lung tissue. While degradation and clearance of the SWCNT was not quantified in this study, the images suggest some of the particles may be present in immune cells, but overall, they are not rapidly removed.

### SWCNT modulate the production of cytokines and chemokines in IAV infected mice

**Fig. 7:**
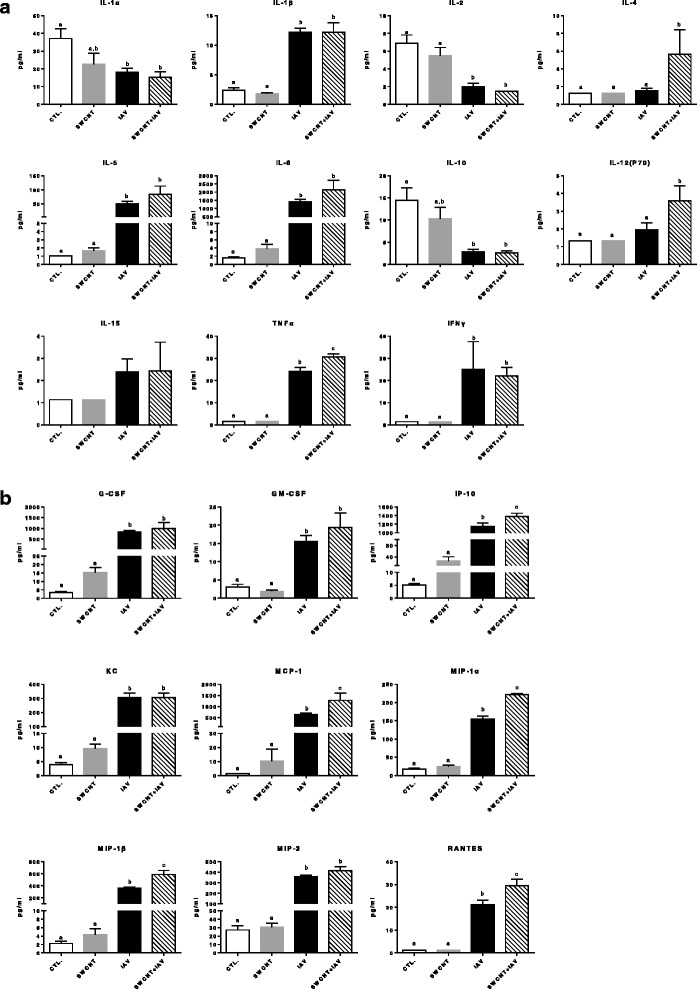
Changes in cytokine and chemokine production in BALF from mice exposed to SWCNT and IAV. C57BL/6 mice were exposed to SWCNT (20 μg per mouse) on day 0 and then to IAV (3.2×10^4^ TCID_50_ per mouse) on day 3. On day 7, mouse lungs were lavaged with PBS and **a** cytokines and **b** chemokines in BALF were measured using multiplex assay. Results are presented as mean±SEM of 6 mice per group. Letters indicate significant differences between groups (*P* < 0.05)

To understand what factors may contribute to the exacerbated IAV titers and inflammation in mouse lungs with SWCNT + IAV exposure, we quantified 25 cytokines and chemokines in BALF. A few of the proteins were not detected in any of the samples (IL-7, IL-9, IL-12(P40), IL-13, IL-17) but most of the cytokines (Fig. 7A) and chemokines (Fig. 7B) measured were significantly increased after IAV infection (IL-1B, IL-5, IL-6, TBFα, IFNγ, G-CSF, GM-CSF, IP-10, KC, MCP-1, MIP-1α, MIP-1β, MIP-2, RANTES), in a manner consistent with previous reports [35, 39, 43–49]. Surprisingly, yet consistent with the BALF cell differentials, SWCNT alone did not elicit robust changes in cytokine or chemokine levels despite the inflammation and injury observed in lung tissues (histopathology), although there were increasing trends for a few targets (IL-6, G-CSF, IP-10, KC, MCP-1, MIP-1β). We also observed several significantly decreased cytokines by IAV infection, including IL-1α, IL-2, and IL-10. With co-exposure of SWCNT + IAV, some cytokines and chemokines were significantly increased in the BALF compared to the IAV only response and included IL-4 (although these levels were low overall), IL-12(p70), TNFα, IP-10, MCP-1, MIP-1α, MIP-1β, and RANTES.

### SWCNT and IAV induced changes in gene expression

**Fig. 8:**
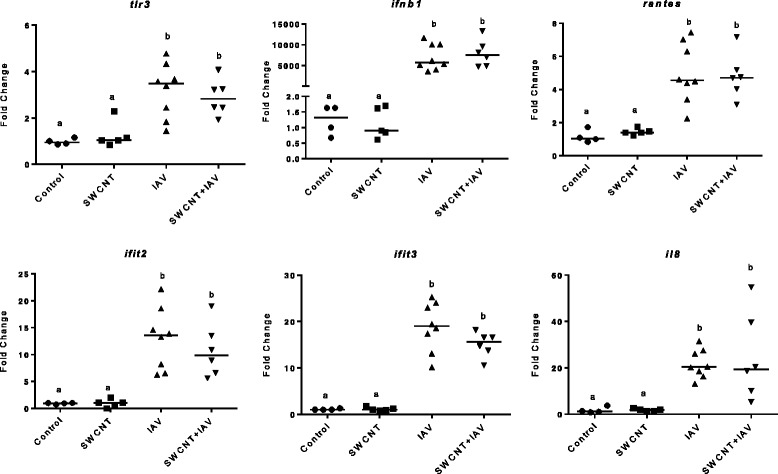
Changes in mRNA expression of antiviral and pro-inflammatory genes in lung tissue from mice exposed to SWCNT and IAV. C57BL/6 mice were exposed to SWCNT (20 μg per mouse) on day 0 and then to IAV (3.2×10^4^ TCID_50_ per mouse) on day 3. On day 7 mouse lungs were homogenized and RNA extracted for qPCR assay. Results are presented as mean±SEM of 4–8 mice per group. Letters indicate significant differences between groups (*P* < 0.05)

To further investigate immune pathways relevant to cellular viral defense mechanisms we examined mRNA expression of several genes in mouse lung tissue including toll-like receptor 3 (*tlr3*), interferon beta 1 (*ifnβ1*), *rantes*, interferon-induced protein with tetratricopeptide repeats 2 (*ifit2*), *ifit3*, and interleukin 8 (*il8*) (Fig. 8). These particular genes were chosen as they are commonly induced by viral infections [15, 16]. Interestingly, we observed a decreasing trend in mRNA expression of *tlr3*, *ifit2*, and particularly *ifit3* in animals exposed to SWCNT + IAV compared to IAV alone.

## Discussion

Here we report, for the first time, that pre-exposure of mice to SWCNT causes a significantly enhanced influenza virus titer in lung tissues. This observation is consistent with results of our previous in vitro study [15] where a significant increase in quantified virus was observed in lung epithelial cells pre-exposed to SWCNT prior to IAV infection. Several other in vivo studies have investigated the ability of chemicals or particulates to alter viral titers but results have been variable between agents. For example, geogenic PM_10_ exposure increased viral titers of influenza H3N1, leading to exacerbated inflammation and lung injury in BALB/c mice [36]. Similarly, exposure to combustion derived PM resulted in the production of oxidative stress accompanied by increased influenza H1N1 infection with suppressed adaptive immune responses in neonatal rats [50]. In contrast, exposure to diesel exhaust exacerbated lung inflammation and injury in mice in response to subsequent IAV infection without significantly increasing viral titers [51, 52] and a similar result was also observed for exposure to cigarette smoke [35, 45]. Consistent across these studies is the noted enhanced lung inflammation with pre-exposure to the particulates prior to viral infection despite the varied increase in titers. This may be partially explained by inherent differences in particulate properties and viruses, exposure times and virus quantitation methods. It is also likely that the particulates vary in their mechanisms of action at the molecular level, a notion that has not been extensively probed.

As expected, exposure to IAV alone resulted in an expected immune response whereby increased cell numbers, particularly neutrophils, were noted in BALF, numerous cytokines and chemokines were significantly increased, and tissues presented with severe interstitial pneumonia, infiltration of lymphocytes and neutrophils, bronchial lesions, and alveolar damage with edema, consistent with previous reports [35, 39, 43–49]. We also observed several significantly decreased cytokines by IAV infection, including IL-1α, IL-2, and IL-10, which vary in their repose to respiratory viruses in the literature. The levels of IL-1α has been shown to both increase and decrease in response to IAV infection [39, 44], which is likely a result of different virus strains as reported for H1N1 A/Cal/04/2009 vs. H1N1 A/Brisbane/59/2007. IL-2 and IL-10 levels were significantly decreased by IAV treatment in our study whereas other findings found no change in mouse BALF in response to IAV [39, 44, 45, 47]. Such discrepancies among studies might come from different virus strains, different time-points, and sensitivity of different protein assays.

Several anti-viral genes were also highly induced by IAV which supports the known function of these proteins in early host immune anti-viral defense mechanisms. For example, as a pattern recognition receptor (PRR), *tlr3* senses double stranded viral RNA which initiates a series of downstream events that include activation of transcription factors NF-kβ and/or interferon regulatory factor (IRF) [53]. These pathways lead to increased transcription of pro-inflammatory and anti-viral genes that include *ifnβ1, Il-8, rantes, ifit2* and *ifit3* [54]. The IFITs directly inhibit viral replication through multiple mechanisms including decreasing translation initiation, binding uncapped RNA for PRR recognition, and sequestering specific viral RNAs or proteins from functioning normally [55]. These proteins serve as a major anti-viral defense mechanism following infection.

The lack of a robust immune response following exposure to SWCNT alone, with respect to differential cell counts, cytokine and chemokine levels, and gene expression was surprising, although a moderate increase of lymphocytes in BALF was observed. These latter observations are in agreement with several studies showing increases in neutrophil and lymphocyte cell populations in response to SWCNT in animal models [37, 38, 56, 57]. The current literature varies when reporting on modulation of immune markers in SWCNT-exposed mice; only some studies have indicated significantly increased BALF cytokine (IL-1β, IL-4, IL-5, IL-6, IL-12) or chemokine levels (G-CSF, MIP-1, MCP-1, KC) [22, 38, 56]; while other reports showed no modulation of these, or other mediators [58]. It has been suggested that nanotubes localize not with macrophages, but with CD11c positive dendritic cells, resulting in suppression of T cell responses [33]. A similar result was also observed in mice exposed to multi-walled carbon nanotubes (MWCNT) that were subsequently stimulated with a mitogen [59]. Interestingly, histopathological changes were quite evident in the SWCNT exposed group despite the lack of immune response (cytokines, genes) which presented as localized pneumonia, observations in line with reports showing that SWCNT induced pneumonitis with accumulation of pulmonary neutrophils and macrophages at peribronchiolar regions and alveoli in conjunction with fibrotic and granulomatous lesions 7 days post exposure [22, 37, 38].

We report several novel observations related to the combined SWCNT + IAV exposure group. First, a mixed immune cell profile was quantified in BALF which consisted of a significant increase in the number of neutrophils (IAV), lymphocytes (SWCNT) and several cytokines and chemokines. For example, there were significantly higher levels of MIP-1α and MIP-1β in the co-exposure group compared to IAV alone, which are known to be important chemotactic and pro-inflammatory factors that activate and recruit granulocytes, such as neutrophils, to sites of infection [60]. Other enhanced immune proteins included IL-4 and IL-12 (p70) which are actively involved in T cell differentiation, activation or maturation while IP-10, MCP-1, and RANTES are chemoattractants that recruit T cells, among others, to sites of infection [44, 45, 47]. TNFα is also an important mediator of fever and systemic inflammation [47] and was also significantly increased in the co-exposure group. Such high levels of cytokines and chemokines paralleled with increased numbers of total immune cells in BALF and exacerbation of tissue inflammation indicating a more than additive immune response for many of the targets measured.

Second, examination of the tissues showed overlap in the nature of the lesions induced by the combined injury yet NIRF imaging analysis showed that the injury was not always affecting the same locations. This is consistent with notion that there was overlap in the nature of the lesions induced by the combined injury yet considerable differences in the magnitude of the lesions. While the mechanisms driving the overlapping yet unique injury profiles of SWCNT and IAV are not yet clear, a few reports have shown CNTs were also able to impair bacterial clearance and phagocytosis that led to worsened infection [2, 22]. A properly regulated immune response is essential for developing an effective defense that includes clearing pathogens and rebuilding damaged tissues [45]; however excessive inflammation can be problematic and detrimental [61, 62]. The enhanced immune response by SWCNT + IAV may dysregulate typical immune responses to IAV infection, leading to increased viral replication.

Third, imaging analysis supports that the SWCNT are not readily cleared. There are several reports that discuss the degradation of SWCNT by immune cell enzymes such as myeloperoxidase (MPO) and nicotinamide adenine dinucleotide phosphate-oxidase (NADPH oxidase) [63, 64]. Interestingly, the degradation capacity of such enzymes varies depending on the SWCNT surface chemistry (pristine vs. oxidized) and coating (i.e. pluronic-PEG, bovine serum albumin (BSA), sodium dodecyl cholate (SDC)) and in fact, one report [65] showed that pluronic-PEG wrapped pristine SWCNT were effectively protected from degradation by MPO. As our SWCNT were prepared in pluronic, they may be less prone to degradation and clearance compared to other types and suspensions of nanotubes.

Fourth, the decreasing trends in mRNA expression of *tlr3*, *ifit2*, and *ifit3* in lung tissue supports a repression of targeted anti-viral defenses which has only recently been reported by our group based on in vitro studies, although the repression was not as strong in vivo. There are likely several reasons that contribute to the lack of substantial repression in in vivo. These include: the expression changes of these genes might be cell-type specific (i.e. epithelial cells) and measurements of whole tissues may mask differences; we only analyzed one series of exposures, SWCNT for 3 days followed by IAV infection for 4 days, and it is possible that at earlier time-points, a greater difference could be observed due to the transient nature of transcription; moderate repression of these virus-responsive genes by SWCNT might still have a significant biological consequence on the cellular anti-viral defense mechanisms, which may partially explain the increased viral titers in the SWCNT + IAV exposed group.

## Conclusions

Research directed at advancing our understanding of SWCNT pulmonary toxicity has shown these particles can induce robust inflammation, fibrosis, granulomas, and pre-cancerous lesions [37, 56, 65–67], but few have studied their influence on pathogen susceptibility. As a follow-up to our previous in vitro study, the present work confirms that SWCNT can increase IAV titers in vivo in concert with exacerbation of inflammation in lung tissues and mechanisms pointing towards modulation of key antiviral and inflammatory markers. Future studies should consider deeper mechanistic probing to elucidate a role for anti-viral proteins, such as IFITs, in SWCNT-induced lung toxicity.

## Additional file

Additional file 1: Table S1A. All mouse weight during 7-day experiment in grams (Mean ± SD). **S1B.** All mouse weight loss during 7-day experiment as % change from day 0 (Mean ± SD). **Table S2.** Primer sets used in this study. ** Figure. S1.** Characterization of SWCNT. Hydrodynamic radii plot of SG65i dialyzed SWCNT used in all experiments. All experiments were performed at 37 ^o^C. **Figure S2.** NIRF of lung tissues among animals in control and IAV only group. Mouse lung H&E stained tissue sections from a (A) control and (B) IAV exposed animal and corresponding NIRF image, 400X. (PDF 879 kb)
