# Supplementary material for: Single-walled carbon nanotubes modulate pulmonary immune responses and increase pandemic influenza a virus titers in mice
Source: Virol J. 2017 Dec 22;14:242. doi: 10.1186/s12985-017-0909-z (PMC5741862; doi:10.1186/s12985-017-0909-z)
Supplement: Additional file 1: Table S1A. — All mouse weight during 7-day experiment in grams (Mean ± SD). S1B. All mouse weight loss during 7-day experiment as % change from day 0 (Mean ± SD). Table S2. Primer sets used in this study. Figure. S1. Characterization of SWCNT. Hydrodynamic radii plot of SG65i dialyzed SWCNT used in all experiments. All experiments were performed at 37 oC. Figure S2. NIRF of lung tissues among animals in control and IAV only group. Mouse lung H&E stained tissue sections from a (A) control and (B) IAV exposed animal and corresponding NIRF image, 400X. (PDF 879 kb) [file 12985_2017_909_MOESM1_ESM.pdf]

Supplemental table 1

Table A: All mouse weight during7-day experiment in grams (Mean ±SD)

Treatment	Days							
	0	1	2	3	4	5	6	7
Control	26.95 ± 2.07	25.27 ± 1.82	25.20 ± 1.65	25.59 ± 1.64	24.88 ± 1.49	25.03 ± 1.61	25.37 ± 1.52	25.79 ± 1.83
SWCNT	25.44 ± 1.85	24.05 ± 2.23	24.72 ± 1.88	24.31 ± 2.21	24.32 ± 1.87	24.46 ± 1.74	25.16 ± 1.75	25.02 ± 1.69
IAV	25.98 ± 1.93	24.88 ± 1.69	24.70 ± 1.89	24.90 ± 1.83	24.12 ± 1.56 [*]	24.20 ± 1.30 [*]	23.33 ± 1.87 [*, #, a]	23.06 ± 1.67 [*, #, a, b, c, d]
SWCNT+IAV	26.58 ± 1.30	25.23 ± 1.36	25.26 ± 1.33	25.70 ± 1.35	25.70 ± 1.48	25.63 ± 1.46	25.31 ± 1.86 [$]	24.44 ± 2.07

Table B: All mouse weight loss during7-day experiment as % change from day 0 (Mean ±SD)

Treatment	Days						
	1	2	3	4	5	6	7
Control	6.18 ± 1.75	6.38 ± 2.46	4.94 ± 2.13	7.53 ± 2.82	7.00 ± 2.58	5.71 ± 2.93	4.23 ± 2.04
SWCNT	5.56 ± 3.46	2.83 ± 3.52	4.52 ± 3.32	4.39 ± 2.80[*]	3.12 ± 1.94 [*]	1.06 ± 2.57 [*, b]	1.61 ± 2.73 [b]
IAV	4.15 ± 3.53	4.91 ± 2.59	4.11 ± 3.26	6.31 ± 3.45	5.90 ± 3.90 [#]	9.46 ± 3.73 [*, #, b, c, d, e, f]	10.41 ± 4.14 [*, #, b, c, d, e, f]
SWCNT+IAV	5.04 ± 3.03	4.94 ± 2.72	3.31 ± 2.06	3.35 ± 1.90[*, $]	3.60 ± 1.82 [*]	4.87 ± 3.20 [#, $]	8.17 ± 4.27 [*, #, b, c, d, e, f]

"*" indicates statistically significant change from control group within each time point; "#" indicates statistically significant change from SWCNT group within each time point; "$"indicates statistically significant change from IAV group within each time point. "a" indicates statistically significant change from day 0 group within each treatment group; "b" indicates statistically significant change from day 1 group within each treatment group; "c" indicates statistically significant change from day 2 group within each treatment group; "d" indicates statistically significant change from day 3 group within each treatment group; "e" indicates statistically significant change from day 4 group within each treatment group; "f" indicates statistically significant change from day 5 group within each treatment group. (P<0.05)

Supplement table 2

Gene name	Forward primer (5’-3’)	Reverse primer (5’-3’)
gapdh	AGG TCA TCC CAG AGC TGA ACG	CAC CCT GTT GCT GTA GCC GTA T
tlr3	ACC TTT GTC TTC TGC ACG AAC CT	AGT TCT TCA CTT CGC AAC GCA
ifnβ1	GGCTTCCATCATGAACAACAGGT	AGGTGAGGTTGATCTTTCCATTCAG
ifit2	CGGAAAGCAGAGGAAATCAA	TGAAAGTTGCCATACCGAAG
ifit3	GACGATTAACGATGGAGTTC	GGGCTCTCCTTACTGATGAC
rantes	GCTGCTTTGCCTACCTCTCC	TCGAGTGACAAACACGACTGC
il8	CTTGAAGGTGTTGCCCTCAG	TGGGGACACCTTTTAGCATC

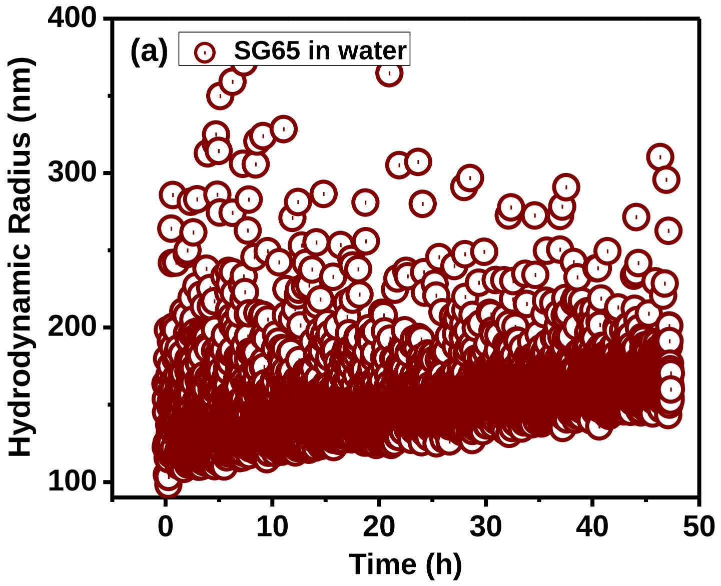

Supplemental Figure 1. Characterization of SWCNT. Hydrodynamic radii plot of SG65i dialyzed SWCNT used in all experiments. All experiments were performed at 37 ^{o}C.

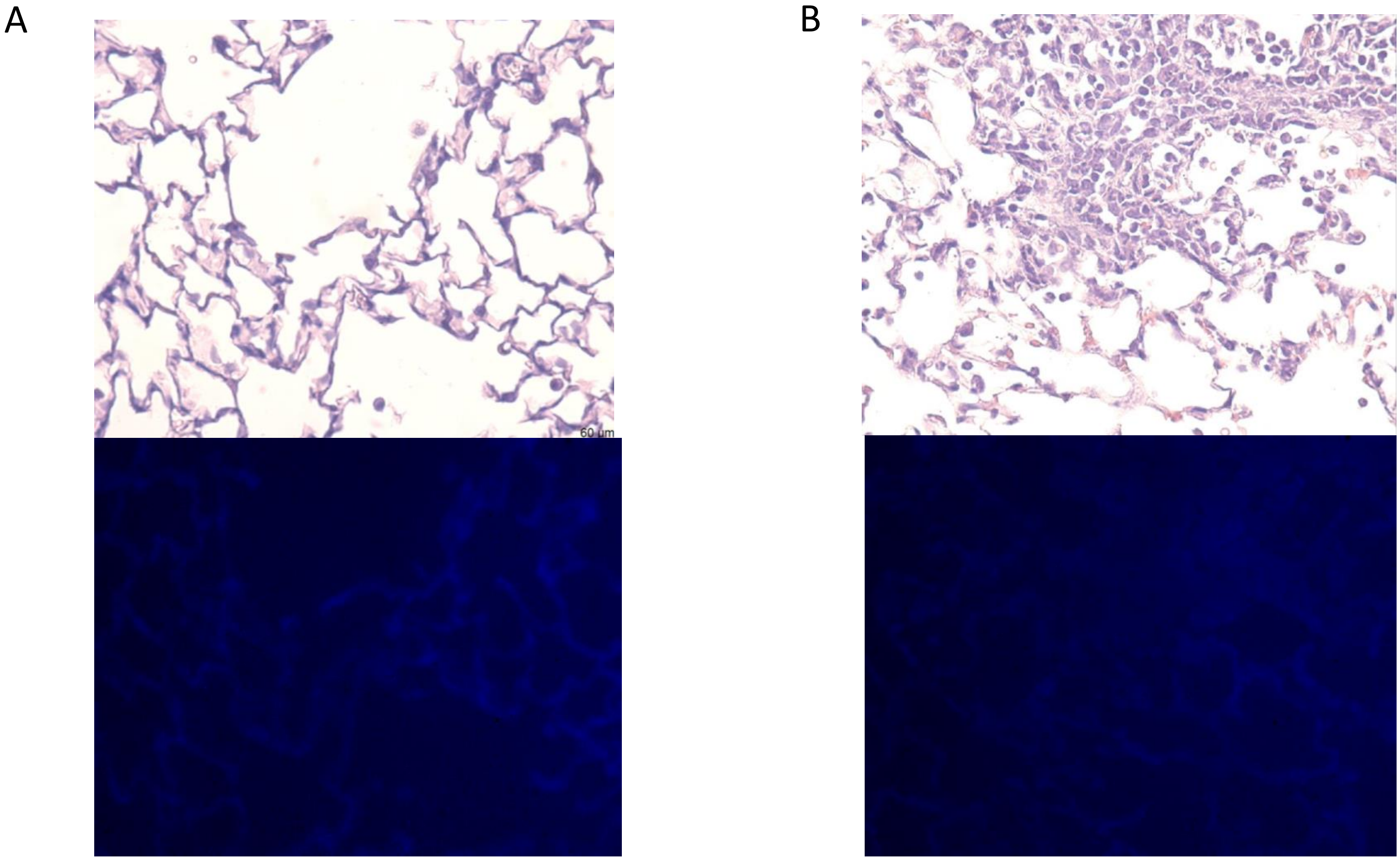

Supplemental Figure 2. NIRF of lung tissues among animals in control and IAV only group. Mouse lung H&E stained tissue sections from a (A) control and (B) IAV exposed animal and corresponding NIRF image, 400X.
